# Supplementary material for: Forensic Application of Stable Isotopes to Distinguish between Wild and Captive Turtles
Source: Biology (Basel). 2022 Nov 29;11(12):1728. doi: 10.3390/biology11121728 (PMC9775157; doi:10.3390/biology11121728)
Supplement: Supplementary file 1 [file biology-11-01728-s001.zip › SupplementalMaterial.pdf]

SUPPLEMENTAL MATERIAL

Predictors	Coefficients
Female	-1.9019160
Male	-0.7525617
$\delta^{13}C$	-4.7990883
$\delta^{15}N$	-1.7239136

Table S1. Coefficients for each predictor in our top model.

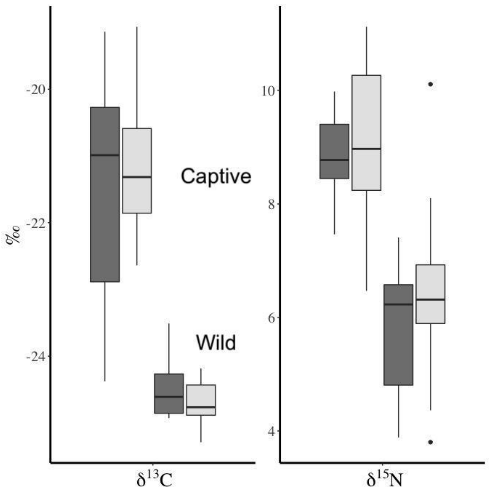

Figure S1. Carbon ($\delta^{13}C$) and nitrogen ($\delta^{15}N$) stable isotope values (per mil, ‰) for the claw tips of female (dark) and male (light) wild wood turtles from Maine and captive wood turtles sampled at various animal care facilities throughout the eastern United States (Table 1).

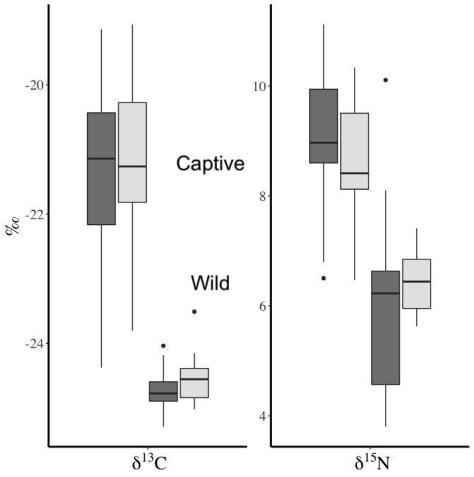

Figure S2. Carbon ($\delta^{13}C$) and nitrogen ($\delta^{15}N$) stable isotope values (per mil, ‰) for the claw tips of wild and captive wood turtles sampled in the fall (dark) and spring (light) (Table 1).
